# Reactive oxygen species are involved in regulating α_1_-adrenoceptor-activated vascular smooth muscle contraction

**DOI:** 10.1186/1423-0127-17-67

**Published:** 2010-08-23

**Authors:** Ming-Ho Tsai, Meei Jyh Jiang

**Affiliations:** 1 Institute of Basic Medical Sciences, College of Medicine, National Cheng Kung University, Tainan 70101, Taiwan; 2 Department of Cell Biology and Anatomy, College of Medicine, National Cheng Kung University, Tainan 70101, Taiwan; 3 Cardiovascular Research Center, College of Medicine, National Cheng Kung University, Tainan 70101, Taiwan

## Abstract

**Background:**

Reactive oxygen species (ROS) were shown to mediate aberrant contractility in hypertension, yet the physiological roles of ROS in vascular smooth muscle contraction have remained elusive. This study aimed to examine whether ROS regulate α_1_-adrenoceptor-activated contraction by altering myosin phosphatase activities.

**Methods:**

Using endothelium-denuded rat tail artery (RTA) strips, effects of anti-oxidants on isometric force, ROS production, phosphorylation of the 20-kDa myosin light chain (MLC_20_), and myosin phosphatase stimulated by α_1_-adrenoceptor agonist phenylephrine were examined.

**Results:**

An antioxidant, N-acetyl-L-cysteine (NAC), and two NADPH oxidase inhibitors, apocynin and VAS2870, dose-dependently inhibited contraction activated by phenylephrine. Phenylephrine stimulated superoxide anion production that was diminished by the pretreatment of apocynin, VAS2870, superoxide scavenger tiron or mitochondria inhibitor rotenone, but not by xanthine oxidase inhibitor allopurinol or cyclooxygenase inhibitor indomethacin. Concurrently, NADPH oxidase activity in RTA homogenates increased within 1 min upon phenylephrine stimulation, sustained for 10 min, and was abolished by the co-treatment with apocynin, but not allopurinol or rotenone. Phenylephrine-induced MLC_20 _phosphorylation was dose-dependently decreased by apocynin. Furthermore, apocynin inhibited phenylephrine-stimulated RhoA translocation to plasma membrane and phosphorylation of both myosin phosphatase regulatory subunit MYPT1^Thr855 ^and myosin phosphatase inhibitor CPI-17^Thr38^.

**Conclusions:**

ROS, probably derived from NADPH oxidase and mitochondria, partially regulate α_1_-adrenoceptor-activated smooth muscle contraction by altering myosin phosphatase-mediated MLC_20 _phosphorylation through both RhoA/Rho kinase- and CPI-17-dependent pathways.

## Background

Excessive production of reactive oxygen species (ROS) causes oxidative stress, which represents an important mechanism in the pathogenesis of vascular diseases such as hypertension and atherosclerosis. However, ROS act as intracellular signaling molecules mediating various cellular functions including proliferation, apoptosis and survival [1]. Emerging evidence also indicated that ROS can regulate vasoconstriction or vasodilatation depending on the vascular bed studied and oxygen radicals formed [2]. Superoxide anion (·O_2_^-^) was shown to mediate hypertension induced by vasoactive factors such as angiotensin II [3,4] and endothelin [5] or by deoxycorticosterone acetate-salt [6]. In addition, superoxide anion amplifies allergen-induced airway hypercontractility [7]. How superoxide anion accomplishes these effects remains poorly understood.

In the vasculature, the potential sources of ROS include NADPH oxidase, uncoupled endothelial nitric oxide synthase, xanthine oxidase, cyclooxygenase and the mitochondrial respiratory chain. Among these, NADPH oxidase is generally considered the major source of vascular ROS [8] and has been shown to regulate myogenic constriction [9] and endothelin 1-activated vascular tone [10]. However, a recent study suggested that mitochondria-derived, not NADPH oxidase-derived, ROS are involved in agonist-stimulated vasoconstriction [11].

Phosphorylation of the 20-kDa myosin light chains (MLC_20_) is a key determinant for smooth muscle contraction. The levels of MLC_20 _phosphorylation are determined by the activity ratio between myosin light chain kinase (MLCK) and myosin phosphatase. While MLCK activation depends on the cytoplasmic calcium concentration, myosin phosphatase activity is subject to the modulation by various signaling molecules [12]. Myosin phosphatase is a heterotrimer consisting of a 37- to 38-kDa catalytic subunit, PP1δ, a 110- to 130-kDa regulatory subunit referred to as myosin phosphatase targeting subunit 1 (MYPT1), and a 20-kDa subunit. Multiple vasoconstrictors inhibit myosin phosphatase activities through the phosphorylation of MYPT1 and/or an endogenous myosin phosphatase inhibitor CPI-17 [13]. *In vivo *evidence showed that Rho kinase plays important roles in MYPT1 phosphorylation whereas protein kinase C catalyzes CPI-17 phosphorylation [13,14].

Recent evidence indicated that ROS mediate α_1_-adrenoceptor-stimulated hypertrophy of vascular smooth muscle and cardiomyocytes, a long-term effect of catecholamines [15-17]. Currently, the contribution of ROS to the acute vasoconstrictor effect of α_1_-adrenoceptors has not been characterized. ROS generated exogeneously by xanthine oxidase activate Rho/Rho kinase-mediated Ca^2+ ^sensitization pathway to contract rat aorta [18]. Our previous study showed that α_1_-adrenoceptor stimulation activates Rho kinase-mediated MYPT1 phosphorylation and protein kinase C-mediated CPI-17 phosphorylation to regulate vasoconstriction [19]. Whether ROS regulate vasoconstrictors-activated contractile force and MLC_20 _phosphorylation by altering myosin phosphatase activities remains unclear. Therefore, this study investigated whether α_1_-adrenoceptor activation triggers ROS formation to regulate contraction through altering myosin phosphatase activity.

## Materials and methods

### Tissue preparation and isometric force measurement

This study conforms to the procedures described in the *Guide for the Care and Use of Laboratory Animals *of the National Institute of Health (U. S. A.), and the experimental procedures were approved by the institutional Animal Care and Use Committee. Male Sprague-Dawley rats weighing 400 ~ 550 g were used in this study. After the animal was anesthetized with pentobarbital (60 mg kg^-1^, i.p.), the tail artery was removed and placed in oxygenated (95% O_2 _- 5% CO_2_) Krebs' physiological salt solution (PSS) with the following composition (in mM): 120 NaCl, 5.9 KCl, 25 NaHCO_3_, 1.2 NaH_2_PO_4_, 11.5 dextrose, 1.2 MgCl_2 _and 2.5 CaCl_2 _. The endothelium-denuded rat tail artery (RTA) strips were placed in tissue bathes with one end held in a muscle holder and the other end connected to a force transducer. After being stretched to the length that allows for maximal force production and being equilibrated at 37°C for at least 1 h, muscle strips were stimulated twice with 51 mM KCl-PSS (equimolar replacement of NaCl with KCl) to generate reproducible contraction. A dose response was generated with cumulative concentrations of α_1_-adrenoceptor agonist phenylephrine and the maximal force was used to normalize later contractile responses. To determine the involvement of ROS and NAD(P)H oxidase, tissues were incubated for 30 min in PSS containing vehicle, N-acetyl-L-cysteine (NAC), apocynin or VAS2870. A cumulative concentration-response for phenylephrine was then performed again. In all other experiments, 10 μM phenylephrine was used.

### Measurement of superoxide anion production

Superoxide production in RTA strips was measured by a lucigenin-enhanced chemiluminescence assay according to a published method [20]. RTA strips 10 mm long were equilibrated in modified PSS-HEPES buffer (in mM: 119 NaCl, 20 HEPES, 4.6 KCl, 1 MgSO_4_, 0.15 Na_2_HPO_4_, 0.4 KH_2_PO_4_, 5 NaHCO_3_, 5.5 glucose, 1.2 CaCl_2_, pH7.4) at 37°C for 30~60 min. After equilibration, RTA strips were incubated for 30 min in prewarmed PSS-HEPES buffer containing 10 mM DETC (Sigma) in the presence or absence of inhibitors for superoxide generation. DETC was used to inhibit the endogenous Cu^2+^/Zn^2+ ^superoxide dismutase (SOD) activity. Following DETC treatment, RTA strips were placed in temperature-controlled (37 °C) sample dishes containing 2 ml HEPES buffer with lucigenin (20 μM, Sigma) plus or minus inhibitors and placed in the dark chamber of the Chemiluminescence Analyzing System (Tohoku Electronic Industrial Co., Japan). The background counts were measured for 5 min, phenylephrine in 100 μl HEPES buffer was then injected into the chamber and the chemiluminescence was measured for another 10 min. Lucigenin chemiluminescence was expressed as counts per min (cpm) per milligram tissue weight after subtracting background counts (without vessel).

### Measurement of Vascular NAD(P)H Oxidase activity

RTA strips were stimulated with phenylephrine, snap-frozen in liquid nitrogen at designated time points, and stored at - 80 °C until use. NAD(P)H oxidase activity was assayed according to a published method [21]. Briefly, RTA strips were homogenized on ice with a glass-to-glass homogenizer for 2 min in phosphate buffer (20 mM K_2_HPO_4_, pH 7.4, 10 μg ml^-1 ^aprotinin, 10 μg ml^-1 ^leupeptin, and 0.5 mM phenylmethylsulfonyl fluoride). Following 10-min centrifugation at 1,000 g, supernatant (30-40 μl) was mixed with reaction buffer containing (in mM) 37 NaCl, 2.7 KCl, 4.3 Na_2_HPO_4_, 1.5 KH_2_PO_4_, and 5 μM lucigenin as the detector. The reaction was started by the addition of NADPH (300 μM, Sigma) and photon emission was measured for 10 min at room temperature in a scintillation counter (LS-6500, Beckman) in out-of-coincidence mode. NADPH oxidase activity was expressed as cpm per microgram protein. The specificity of lucigenin for ·O_2_^- ^production was validated by adding a nonenzymatic scavenger of ·O_2_^-^, tiron (10 mM, Sigma), to quench the signal. To examine the effects of inhibitors, 1 mM apocynin, 10 μM VAS2870, 100 μM allopurinol, 10 μM rotenone or vehicle was added 30 min before RTA strips were stimulated with phenylephrine for 1 min.

### Measurement of MLC_20 _phosphorylation

MLC_20 _phosphorylation was measured by two-dimensional polyacrylamide gel electrophoresis (PAGE) as previously described [19]. Briefly, homogenates of RTA strips were sequentially resolved by isoelectric focusing gel electrophoresis (Pharmalytes of 80% pH 4.5-5.4 and 20% pH 3-10, Amersham Pharmacia Biotech) in the first dimension and SDS-PAGE in the second dimension. The gels were silver stained and analyzed by densitometry. Data are expressed as the ratio of phosphorylated MLC_20 _over total (phosphorylated plus unphosphorylated) MLC_20_.

### Western blot analysis of MYPT-1 and CPI-17 phosphorylation levels

Equal amounts of protein were resolved under reducing conditions on 8% SDS-PAGE (for MYPT1) or 12.5% SDS-PAGE (for CPI-17) and transferred electrically onto nitrocellulose membranes. Following 1.5 h blocking in a Tris-buffered saline (TBS) solution containing 3% nonfat dry milk at room temperature, membranes were incubated overnight at 4°C with phosphospecific anti-MYPT1^Thr697 ^or anti-MYPT1^Thr855 ^antibody (1:1000, Upstate) or anti-CPI-17^Thr38 ^antibody (1:500, Upstate). The blots were washed and incubated with horseradish peroxidase-conjugated anti-rabbit IgG (1:5000, Chemicon) for 1.5 h at room temperature. Immunoreactive bands were visualized by enhanced chemiluminescence (PerkinElmer Life Sciences). Membranes were then stripped of bound antibodies by incubation in a buffer containing 62.5 mM Tris-HCl (pH 6.8), 2% SDS, and 10 mM DTT for 30 min at 50°C with agitation. The blots were reprobed with anti-MYPT1 (1:7500, Covance) or anti-CPI-17 (1:2000, Upstate) for loading control.

### RhoA translocation assay

Translocation of RhoA from the cytosol to membrane fractions was determined according to a published method [22] with modifications. RTA strips stimulated with phenylephrine for 1 min or 15 min with or without inhibitors were snap frozen in liquid nitrogen. After RTA strips were homogenized in ice-cold buffer (in mM: 50 HEPES, pH 7.5, 50 NaCl, 1 MgCl_2_, 2 EDTA, 10 μg ml^-1 ^aprotinin and leupeptin, 1 phenylmethylsulfonyl fluoride, 50 sodium orthovanadate, 10 pyrophosphate, 10 NaF), the homogenates were centrifuged at 100,000 g, 4 °C for 30 min (Beckman Optima TLX Ultracentrifuge, TLA 120.2 rotor). The supernatant was collected as cytosolic fraction, and membrane proteins were extracted by incubating the pellet for 30 min in homogenization buffer containing 1% Triton X-100 and 0.25% sodium cholate. The extract was clarified at 800 *g *for 10 min, and the supernatant was collected as the particulate fraction. Protein concentrations were determined with a bicinchoninic acid kit (Pierce). Equal amounts of protein were electrophoresed on 12.5% SDS-PAGE followed by immunoblotting with a monoclonal anti-RhoA antibody (1:500, Santa Cruz). The result was expressed as the ratio of membrane RhoA/total RhoA (membrane RhoA + cytosolic RhoA).

### Statistical Analysis

Data were expressed as means ± SEM. For multiple comparisons, ANOVA followed by Newman-Kuels or Dunnett's post-hoc test was used (Prism 4.0; GraphPad Software, San Diego, CA). An unpaired Student's *t *test was used for comparison between control and treatment group. *P*-values less than 0.05 were considered statistically significant.

## Results

### Antioxidant and NADPH oxidase inhibitors decrease phenylephrine-induced force

**Figure 1:**
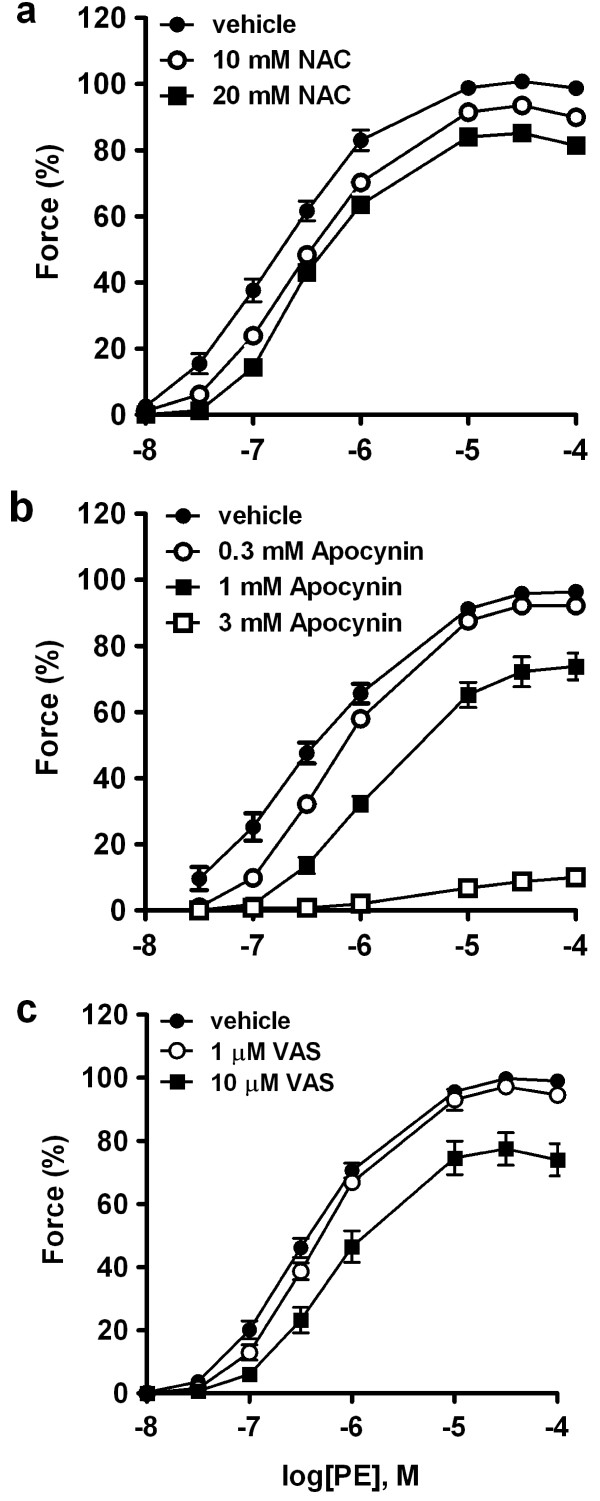
**Effects of NAC (a), apocynin (b) and VAS2870 (c) on phenylephrine-induced contraction of RTA strips**. Two cumulative concentration-responses were constructed in RTA strips stimulated with α_1_-adrenoceptor agonist phenylephrine. For the second contraction, RTA strips were preincubated for 30 min with NAC (10~20 mM), apocynin (0.3~3 mM), VAS2870 (1~10 μM) or vehicle, then were stimulated with agonist in the continuous presence of the inhibitor. Data are mean ± S.E.M. of 6 independent experiments. Force is expressed as the percentage of maximal force obtained in the first contraction.

The involvement of ROS in α_1_-adrenoceptor-activated contraction was assessed by the effects of an antioxidant and two NAD(P)H oxidase inhibitors on phenylephrine-stimulated isometric force of RTA strips. As shown in Figure 1, pretreating RTA with antioxidant NAC (A) or NAD(P)H oxidase inhibitors, apocynin (0.3-3 mM, B) and VAS2870 (1-10 μM, C), caused a rightward shift of the concentration-response curve and reduced the maximal force to phenylephrine. These results suggested that ROS, possibly derived from NADPH oxidase, are involved in phenylephrine-activated contraction.

### Phenylephrine stimulates ROS generation in RTA

**Figure 2:**
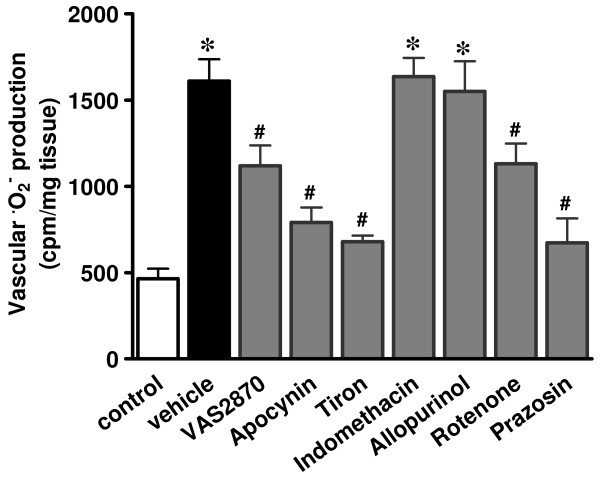
**Phenylephrine-stimulated superoxide anion generation**. RTA strips were stimulated with 10 μM phenylephrine and superoxide anion generation was measured by lucigenin-enhanced chemiluminescence as described in Methods. Some vessels were incubated for 1 hr with α_1_-adrenoceptor antagonist prazosin (1 μM) or for 30 min with VAS2870 (10 μM), apocynin (1 mM), tiron (10 mM), indomethacin (10 μM), allopurinol (100 μM), rotenone (10 μM) or vehicle (DMSO or ethanol) before and during phenylephrine treatment. As the values obtained in the presence of DMSO or ethanol were not different from that of phenylephrine alone, the data were pooled. Each bar represents mean ± S.E.M. of six to ten independent experiments. *p < 0.001 vs. control; ^#^*p *< 0.05 vs. vehicle.

We next examined whether phenylephrine stimulated ROS production in RTA strips using lucigenin-enhanced chemiluminescence assay. As shown in Figure 2, phenylephrine significantly increased chemiluminescence signals compared to those of non-stimulated control (1611 ± 125.9 cpm mg tissue^-1 ^vs. 464.3 ± 58.8 cpm mg tissue^-1^, *p *< 0.05). Pretreatment with a selective α_1_-adrenoceptor antagonist prazosin (1 μM) nearly abolished phenylephrine-stimulated superoxide production (672.0 ± 142.6 cpm mg tissue^-1^) and contractile response (data not shown). This result indicated that phenylephrine stimulates superoxide production in α_1_-adrenoceptor-dependent manner. To further unravel sources for phenylephrine-stimulated superoxide anions, we examined the effect of inhibitors for various ROS generation systems. Phenylephrine-induced ·O_2_^- ^production was inhibited by the pretreatment with NAD(P)H oxidase inhibitors apocynin (1 mM) and VAS2870 (10 μM), a superoxide scavenger tiron (10 mM), and mitochondria respiratory chain inhibitor rotenone (10 μM). In contrast, xanthine oxidase inhibitor allopurinol (100 μM) and cyclooxygenase inhibitor indomethacin (10 μM) had no effect. These results suggested that NAD(P)H oxidase and mitochondria, but not xanthine oxidase or cyclooxygenase, may contribute to phenylephrine-stimulated superoxide production.

**Figure 3:**
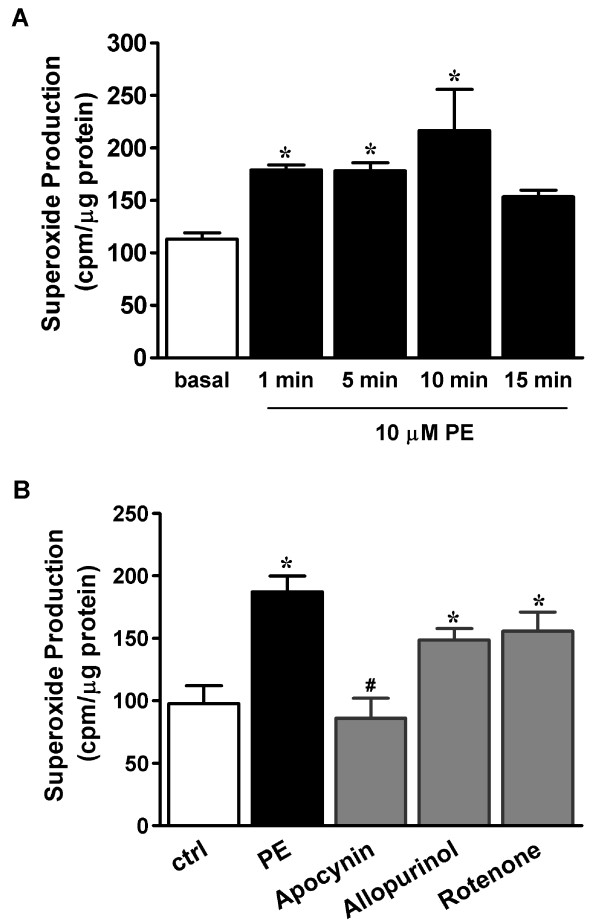
**NADPH oxidase activation in response to phenylephrine stimulation**. (a) Time course. RTA strips were stimulated with 10 μM PE for the indicated period. NADPH oxidase activity was measured in RTA homogenates as described in Methods and was expressed as mean ± SEM of 5 independent experiments. *P< 0.05 vs. unstimulated control. (b) Effects of pro-oxidative enzyme inhibitors. RTA strips were incubated for 30 min with the indicated inhibitors, followed by 1-min phenylephrine stimulation. Results were expressed as mean ± S.E.M. of 6~8 independent experiments. *P < 0.05 vs. unstimulated control; #p < 0.01 vs. phenylephrine alone.

The role of NAD(P)H oxidase in phenylephrine-induced superoxide production was further corroborated by activity assay in RTA homogenates. We tested both NADH and NADPH as the substrate for activity assay and found that 300 μM NADPH, but not NADH, elicited detectable signals. As shown in Figure 3A, activation of NAD(P)H oxidase occurred within 1 min upon phenylephrine stimulation and persisted for at least 10 min (113 ± 6 cpm μg protein^-1 ^for control, 179 ± 5 and 217 ± 32 cpm μg protein^-1 ^for 1-min and 10-min phenylephrine stimulation, respectively, *p *< 0.05 compared to control). As shown in Figure 3B, apocynin (1 mM) effectively reduced phenylephrine-stimulated chemiluminescence at 1 min (185 ± 14 cpm μg protein^-1 ^for vehicle control, 86 ± 16 cpm μg protein^-1 ^for apocynin; *p *< 0.05). In contrast, neither allopurinol (100 μM) nor rotenone (10 μM) exhibited significant inhibition. These results implied that NAD(P)H oxidase is involved in phenylephrine-stimulated superoxide anion production in RTA strips.

### ROS regulate phenylephrine-stimulated MLC_20 _phosphorylation

**Figure 4:**
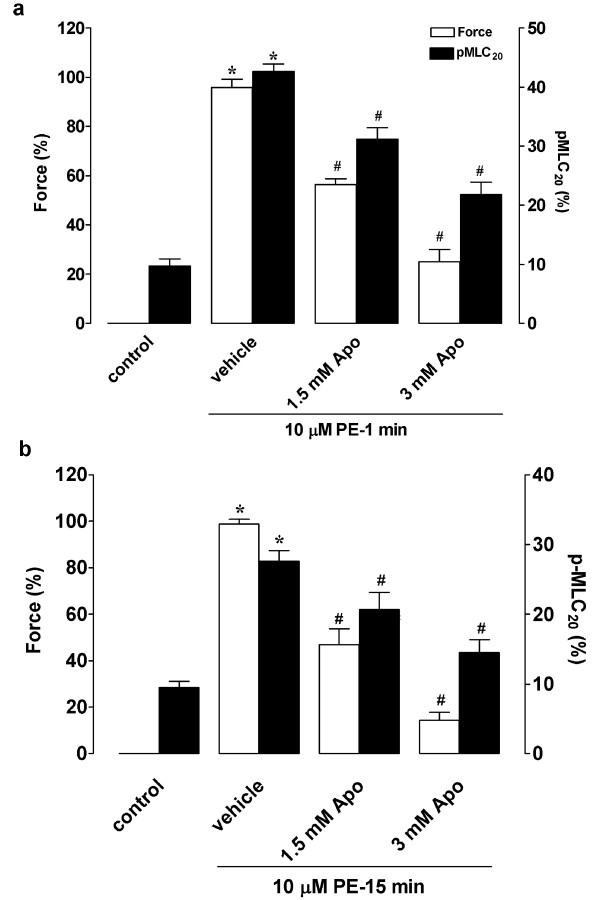
**Effects of apocynin on phenylephrine-induced MLC_20 _phosphorylation**. RTA strips were pretreated for 30 min with apocynin or vehicle, followed by phenylephrine stimulation. Tissues were snap-frozen at 1 min (a) or 15 min (b). MLC_20 _phosphorylation was analyzed by 2-D PAGE as described in Methods. Results are expressed as mean ± S.E.M. from 5~9 independent experiments. Apo: apocynin. * *p *< 0.01 vs. resting control; ^# ^*p*< 0.05 vs. phenylephrine alone.

To determine whether ROS are involved in regulating phenylephrine-activated MLC_20 _phosphorylation, we measured MLC_20 _phosphorylation levels in the presence and absence of apocynin. As depicted in Figure 4A, at 1-min phenylephrine stimulation, MLC_20 _phosphorylation increased markedly from 10 ± 1.1% to 43 ± 1.2% (p < 0.001), pretreatment of apocynin (1.5 or 3 mM) dose-dependently inhibited phenylephrine-stimulated MLC_20 _phosphorylation. Similarly, at 15-min phenylephrine stimulation, MLC_20 _phosphorylation was inhibited by apocynin in a dose-dependent manner (p < 0.05, Figure 4B). These results suggest that ROS modulate phenylephrine-stimulated MLC_20 _phosphorylation.

### ROS regulate phenylephrine-induced MYPT1 phosphorylation

**Figure 5:**
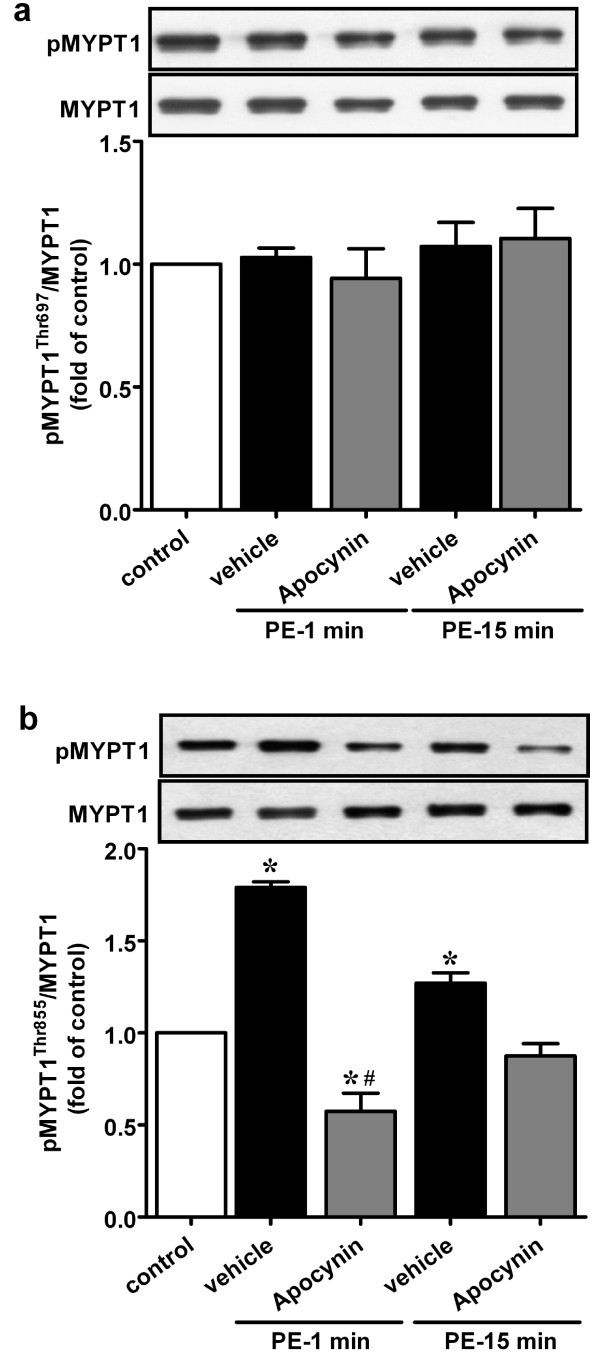
**Effects of apocynin on phenylephrine-induced MYPT1 phosphorylation**. RTA strips were pre-incubated with apocynin or vehicle for 30 min and then challenged with phenylephrine for 1 or 15 min. Phosphorylation levels of MYPT1 at Thr697 (a) and Thr855 (b) were assessed by immunoblotting using phospho-specific MYPT1 antibody. Equal protein loading was verified by total MYPT1 immunoreactivity. The upper panels show representative immunoblots and the lower panels summarize the densitometric results. Values were normalized against control and data are presented as mean ± S.E.M. of four independent experiments. *p < 0.05 vs. control; #*p *< 0.05 compared with time-matched phenylephrine alone.

To elucidate whether ROS affect MLC_20 _phosphorylation through inhibiting myosin phosphatase, MYPT1 phosphorylation levels at two regulatory sites, Thr697 and Thr855, were examined. As shown in Figure 5B, within 1 min of phenylephrine stimulation, MYPT1^Thr855 ^phosphorylation increased approximately 2-fold and was eliminated with apocynin pretreatment. At 15 min, phenylephrine caused a small but significant increase in MYPT1^Thr855 ^phosphorylation, which was attenuated by apocynin. In contrast to MYPT1^Thr855 ^phosphorylation, no change was detected in MYPT1^Thr697 ^phosphorylation in response to phenylephrine or apocynin (Figure 5A).

### ROS regulate phenylephrine-induced RhoA translocation

**Figure 6:**
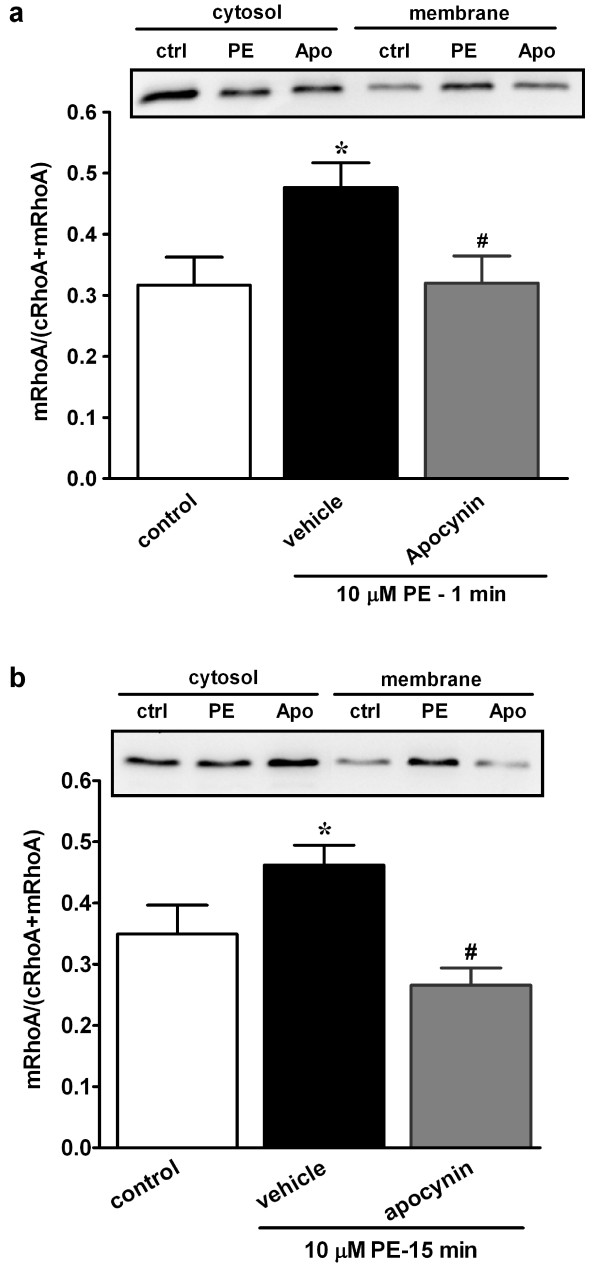
**Effects of apocynin on phenylephrine-induced RhoA membrane translocation**. RTA strips were pre-incubated with apocynin or vehicle for 30 min and then challenged with phenylephrine for 1 min (a) or 15 min (b). The cytosolic and membrane fractions were separated by ultracentrifugation and RhoA in each fraction were analyzed by immunoblotting as described in Methods. The top panels show representative immunoblots. The bottom panels summarize the quantitative results, expressed as the ratio of membrane RhoA to total RhoA, from six independent experiments. The bars represent means ± S.E.M.. *p < 0.05 vs. control; ^#^*p *< 0.05 vs. phenylephrine alone.

Since phenylephrine-stimulated MYPT1^Thr855 ^phosphorylation is Rho kinase-dependent [19], the effects of suppressing ROS generation on phenylephrine-induced RhoA activation were examined by translocation assay. As illustrated in Figure 6A, the fraction of membrane RhoA (membrane RhoA/total RhoA) in the resting state was 32 ± 4.5% and rapidly increased to 48 ± 4.0% at 1 min after phenylephrine stimulation (*p *< 0.05, *n *= 6). Pretreatment of RTA with apocynin abolished phenylephrine-induced RhoA translocation (32 ± 4.4% for apocynin, *p *< 0.05 compared to phenylephrine alone, *n *= 6). Similar results were obtained at 15-min phenylephrine stimulation (Figure 6B). The results of Figure 5 and 6 suggest that ROS are involved in α_1_-adrenoceptor-stimulated RhoA/Rho kinase activation.

### ROS regulate phenylephrine-stimulated CPI-17 phosphorylation

**Figure 7:**
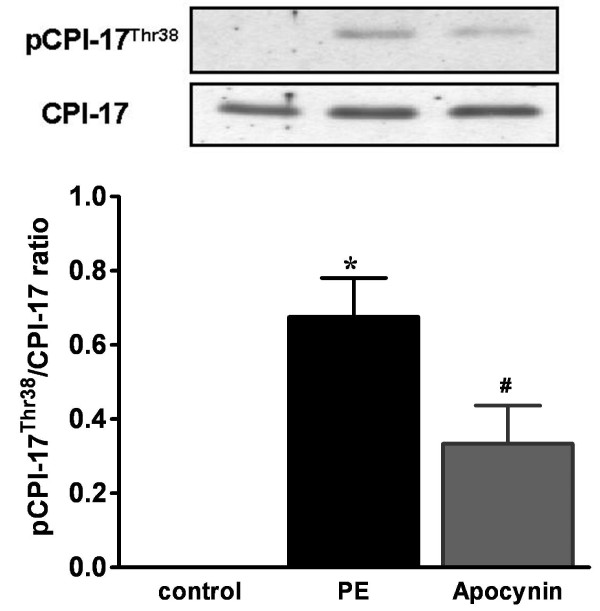
**Effects of apocynin on phenylephrine-induced CPI-17 phosphorylation**. RTA strips were incubated with apocynin (3 mM) or vehicle for 30 min and then stimulated with phenylephrine for 1 min. The tissue was snap-frozen and CPI-17 phosphorylation was assessed with CPI-17^Thr38 ^phospho-specific antibody. Equal protein loading was verified by reprobing for total CPI-17 immunoreactivity. The top panel shows the representative blots of phospho-CPI-17^Thr38 ^and total CPI-17. The bottom panel summarizes the densitometric results. The phosphorylation levels of CPI-17 were quantified as the ratio of phosphorylated CPI-17 to total CPI-17 protein. The bars represent means ± S.E.M. of 9 experiments. *p < 0.05 vs. control; ^#^*p *< 0.05 vs. phenylephrine alone.

Our previous results showed that phenylephrine stimulated CPI-17^Thr38 ^phosphorylation at the initial phase of contraction in RTA strips [19]. Therefore, we examined whether ROS regulate myosin phosphatase activity through CPI-17^Thr38 ^phosphorylation. As shown in Figure 7, CPI-17^Thr38 ^phosphorylation at 1 min of phenylephrine stimulation was markedly inhibited by apocynin pretreatment. This result suggests that CPI-17 phosphorylation also provides a mechanism for ROS to regulate myosin phosphatase activity during α_1_-adrenoceptor activation.

## Discussion

Results of this study show that α_1_-adrenoceptor activation induced ROS (probably ·O_2_^-^) production to regulate vasoconstriction. While inhibition of xanthine oxidase or cyclooxygenase did not affect ROS production in phenylephrine-stimulated RTA, two structurally distinct inhibitors of NAD(P)H oxidase abolished this response. Our results also indicated that phenylephrine-activated contractile response in RTA is only partially regulated by ROS because apocynin and VAS2870, at concentrations that attenuated 50~80% of ROS production, only decreased force by 30%~40%.

Our results showed that α_1_-adrenoceptor activation upon phenylephrine challenging increased ROS production. What is responsible for α_1_-adrenoceptor-mediated vascular ROS generation? Xanthine oxidase and cyclooxygenase can be excluded since inhibiton of either enzyme had no effect on phenylephrine-induced ROS formation. One probable source is NADPH oxidase. Our finding that phenylephrine-induced ROS production was attenuated by apocynin or VAS2870 pretreatment supports this notion. Furthermore, phenylephrine stimulation increased NADPH oxidase activity in RTA homogenate, which was abolished by apocynin. For more than a decade, apocynin has been used as a specific inhibitor to explore the role of NADPH oxidase in the field of vascular biology. However, Heumueller et al recently reported that apocynin is not an inhibitor of vascular NADPH oxidase and instead acts as an antioxidant, thereby reducing ROS bioavailability [23]. These authors found that in the presence of myeloperoxidase (MPO), apocynin is oxidized to form active dimers capable of blocking the assembly and activation of NADPH oxidase. As vascular smooth muscle cells do not express MPO, apocynin may not form active dimers and hence can not block NADPH oxidase activation. Regardless of mechanisms acted by apocynin, a novel NADPH oxidase inhibitor, VAS2870 [24], inhibited phenylephrine-activated force and superoxide production by ~30% in RTA strips. Taken together, NADPH oxidase-derived ROS are likely to be involved in α_1_-adrenoceptor-activated vasoconstriction.

Mitochondria present another likely source for α_1_-adrenoceptor-induced ROS generation. An inhibitor of mitochondrial respiratory chain, rotenone, markedly decreased phenylephrine-induced ROS formation. This finding is in good agreement with a previous study showing that mitochondria are a major source of ROS in response to phenylephrine stimulation in rat mesenteric artery [11]. Using mitochondria-selective ROS scavenger and fluorescent probe, the authors provide convincing evidence to support the essential role of mitochondria-derived ROS in the maintenance of α_1_-adrenergic-dependent vasoconstriction [11]. Another study by Bailey et al also reported that mitochondria-derived ROS in smooth muscle initiate cold-induced constriction of cutaneous arteries [25]. Further experiments are needed to elucidate the underlying mechanisms for mitochondria-derived ROS to regulate α_1_-adrenergic-activated vasoconstriction.

Myosin light chain phosphorylation levels are determined by the activity ratio between myosin light chain kinase and myosin phosphatase. Phosphorylation of MYPT1 inhibits myosin phosphatase activity, resulting in increased MLC_20 _phosphorylation and smooth muscle contraction [26,27]. Results of this study showed that at initial phase α_1_-adrenoceptor stimulation activates NAD(P)H oxidase with concomitant increases in RhoA translocation, MYPT1^Thr855 ^and MLC_20 _phosphorylation. Apocynin at concentrations that abolished increase in ROS production eliminated RhoA translocation and MYPT1^Thr855 ^phosphorylation and decreased MLC_20 _phosphorylation. These results are consistent with the idea that ROS play upstream signaling roles in RhoA/Rho kinase activation and myosin phosphatase inhibition. At sustained phase, similar results were obtained with apocynin on RhoA translocation, MYPT1^Thr855 ^phosphorylation and MLC_20 _phosphorylation. These results suggest that ROS may regulate α_1_-adrenoceptor-stimulated contraction through RhoA/Rho kinase-mediated myosin phosphatase inhibition and thereby increasing MLC_20 _phosphorylation. It was previously reported that exogenously generated ·O_2_^- ^activates Rho/Rho kinase pathway to promote contraction [18]. Knock et al. recently showed that LY83583-generated superoxide activates Rho kinase-mediated phosphorylation of MYPT1^Thr855^, resulting in enhanced MLC_20 _phosphorylation and contraction in rat pulmonary arteries [28]. Up to date, superoxide-dependent Rho/Rho kinase activation in the artery under physiological conditions has only been documented in chronic angiotensin II-induced hypertensive rats [29]. Results of this study provide the first evidence indicating that ROS-mediated RhoA activation occurs under the activation of vasoconstrictor receptors. In this context, RhoA was recently reported to be directly activated by ROS through a redox-sensitive motif involving two cysteines within the phosphoryl binding loop [30], a redox-active domain present in most Rho GTPases [31].

Phosphorylation of CPI-17, a smooth muscle-specific protein inhibitor of myosin phosphatase, by PKC presents another important mechanism to inhibit myosin phosphatase activity [32,33]. It was shown that PKC isoforms, including α, βI, γ, δ, ε, and ζ are activated by tyrosine phosphorylation, which can be regulated by protein-tyrosine phosphatases (PTP) [34]. As PTP are well-established targets of ROS [35], inhibition of PTP by ROS can enhance PKC- and CPI-17-mediated inhibition of myosin phosphatase, resulting in increased MLC_20 _phosphorylation and smooth muscle contraction. The result that α_1_-adrenoceptor-induced CPI-17 phosphorylation was inhibited by apocynin suggested that CPI-17-mediated myosin phosphatase inhibition provides another mechanism accounting for ROS actions. Interestingly, apocynin was recently reported to inhibit Rho kinase without involving vascular NAD(P)H oxidase [36]. Our result that apocynin inhibited both Rho kinase-dependent MYPT-1 phosphorylation and protein kinase C-dependent CPI-17 phosphorylation strongly suggested that apocynin acts as an antioxidant to inhibit RhoA/Rho kinase and protein kinase C.

In addition to modulating myosin phosphatase activity, ROS modulate intracellular Ca^2+ ^mobilization [37]. It was reported that ROS increase cytoplasmic Ca^2+ ^by inhibiting Ca^2+^-ATPase activity and by promoting inositol trisphosphate-induced Ca^2+ ^release [38]. A recent report illustrated that a functional NAD(P)H oxidase system exists in the sarcoplasmic reticulum of coronary artery smooth muscle, which produces ·O_2_^- ^locally to regulate ryanodine receptor activity and Ca^2+ ^release from sarcoplasmic reticulum [39]. Whether ROS also increase MLC_20 _phosphorylation via the activation of MLCK remains to be examined.

In summary, the present study showed that α_1_-adrenoceptor-activated smooth muscle contraction is modulated by ROS generation. Alpha1 adrenoceptor-stimulated increases in ROS production, acting through RhoA/Rho kinase-mediated MYPT1^Thr855 ^phosphorylation and Rho kinase-independent CPI-17^Thr38 ^phosphorylation, lead to myosin phosphatase inhibition, MLC_20 _phosphorylation, and smooth muscle contraction.

## Competing interests

The authors declare that they have no competing interests.

## Authors' contributions

MHT designed and performed the experiments, analyzed the data, and drafted the manuscript; MJJ conceived of the study, designed the experiments, analyzed the data, and helped draft the manuscript. Both authors read and approved the final manuscript.
